# Analysis of proteomic changes in cassava cv. Kasetsart 50 caused by *Sri Lankan cassava mosaic virus* infection

**DOI:** 10.1186/s12870-022-03967-1

**Published:** 2022-12-10

**Authors:** Wanwisa Siriwan, Nuannapa Hemniam, Nattachai Vannatim, Srihunsa Malichan, Somruthai Chaowongdee, Sittiruk Roytrakul, Sawanya Charoenlappanit, Aroonothai Sawwa

**Affiliations:** 1 Department of Plant Pathology, Faculty of Agriculture, Kasetsart University, Bangkok, 10900 Thailand; 2 Center of Excellence On Agricultural Biotechnology (AG-BIO/MHESI), Bangkok, 10900 Thailand; 3 Center for Agricultural Biotechnology, Kasetsart University, Kamphaengsaen Campus, Nakhon Pathom, 73140 Thailand; 4 National Center for Genetic and Engineering and Biotechnology (BIOTECH), National Science and Technology Development Agency, Pathumthani, 12100 Thailand; 5 Biotechnology Research and Development Office, Department of Agriculture, Thanyaburi, Pathumthani, 12110 Thailand

**Keywords:** Proteomic profiling, Cassava cv. Kasetsart 50, Geminivius and Sri Lankan cassava mosaic virus

## Abstract

**Background:**

Sri Lankan cassava mosaic virus (SLCMV) is a plant virus causing significant economic losses throughout Southeast Asia. While proteomics has the potential to identify molecular markers that could assist the breeding of virus resistant cultivars, the effects of SLCMV infection in cassava have not been previously explored in detail.

**Results:**

Liquid Chromatography-Tandem Mass Spectrometry (LC/MS–MS) was used to identify differentially expressed proteins in SLCMV infected leaves, and qPCR was used to confirm changes at mRNA levels. LC/MS–MS identified 1,813 proteins, including 479 and 408 proteins that were upregulated in SLCMV-infected and healthy cassava plants respectively, while 109 proteins were detected in both samples. Most of the identified proteins were involved in biosynthetic processes (29.8%), cellular processes (20.9%), and metabolism (18.4%). Transport proteins, stress response molecules, and proteins involved in signal transduction, plant defense responses, photosynthesis, and cellular respiration, although present, only represented a relatively small subset of the detected differences. RT-qPCR confirmed the upregulation of WRKY 77 (A0A140H8T1), WRKY 83 (A0A140H8T7), NAC 6 (A0A0M4G3M4), NAC 35 (A0A0M5JAB4), NAC 22 (A0A0M5J8Q6), NAC 54 (A0A0M4FSG8), NAC 70 (A0A0M4FEU9), MYB (A0A2C9VER9 and A0A2C9VME6), bHLH (A0A2C9UNL9 and A0A2C9WBZ1) transcription factors. Additional upregulated transcripts included receptors, such as receptor-like serine/threonine-protein kinase (RSTK) (A0A2C9UPE4), Toll/interleukin-1 receptor (TIR) (A0A2C9V5Q3), leucine rich repeat N-terminal domain (LRRNT_2) (A0A2C9VHG8), and cupin (A0A199UBY6). These molecules participate in innate immunity, plant defense mechanisms, and responses to biotic stress and to phytohormones.

**Conclusions:**

We detected 1,813 differentially expressed proteins infected cassava plants, of which 479 were selectively upregulated. These could be classified into three main biological functional groups, with roles in gene regulation, plant defense mechanisms, and stress responses. These results will help identify key proteins affected by SLCMV infection in cassava plants.

**Supplementary Information:**

The online version contains supplementary material available at 10.1186/s12870-022-03967-1.

## Background

After rice and maize, cassava (*Manihot esculenta* Crantz) is the third most widely cultivated crop in the world [1]. Apart from providing food, cassava is also used in the production of starch-based products, animal feed, ethanol, and biofuel. In Southeast Asia [2], cassava is grown as an industrial crop by more than 2 million households. In Thailand alone, 1.28 million hectares of farmland is used for the cultivation of this crop, providing work for more than 600,000 families in 50 provinces. Typically, the country produces 28–30 million tons of casava annually, and demand can exceed 40 million tons [3]. Cassava serves as food and it is also used in the production of starch-based products, animal feed, ethanol, and biofuel. Cultivar Kasetsart 50 (KU50) is one of the most important cassava cultivars in the world, cultivated in several Asian countries. In 2009, the adaptation of KU50 reached its peak; farmers grew this cultivar on 718,400 hectares of land, 52% of this being in Thailand. Its excellent yield, high starch content, good gemination and vigorous growth explain the increasing popularity of this cultivar. It also adapts well to different Agro-ecological zones [4, 5]. Unfortunately, in recent years the production of this valuable crop has been affected by pests and pathogens, reducing productivity.

Cassava mosaic disease (CMD) is the most important plant pest in African and Asia [6]. CMD first emerged in Southeast Asia in 2015 [2, 7]. Currently most CMD outbreaks are reported in mainland SEA, including Cambodia, Vietnam, Thailand, and Laos [7–11]. Uke, et al. reported that cassava root yield decreased by 16–33% and starch content by 22–28%, after infected cuttings were planted in Vietnam [10]. To date, all CMD outbreaks in SEA have been caused by a single viral species, *Sri Lankan cassava mosaic virus* (SLCMV) [12]. Although outbreaks affected SEA for 8 years, currently there are no commercially available SLCMV resistant cassava cultivars. Nonetheless, field trials and greenhouse testing indicated that KU50 showed some tolerance to CMD, with only mildly symptomatic infections and slow disease progression resulting in better preserved yields compared to other commercial cassava varieties [13, 14]. Consequently, the Department of Agriculture Extension, Thailand, has approved KU50 as an acceptable substitute for susceptible cassava varieties until more CMD resistant varieties are developed. SLCMV is a member of cassava mosaic geminiviruses (family Geminiviridae, genus *Begomovirus*). SLCMV is one of eleven cassava mosaic geminivirus species [15] that are primarily transmitted by a whitefly vector (*Bemisia tabaci*), although vegetative propagation also occurs, through the plantation of infected stems [16]. It appears, that the practice of vegetative cassava propagation is the main factor in disease spread in SEA.

Proteomics studies analyze proteins after post-translational modifications (PTMs), providing vital insight into biological functions [17]. PTMs are crucial for plant growth, development, and responses to biotic and abiotic stresses. As PTMs cannot be detected by genome sequencing or transcriptional analysis, proteomics has great utility in understanding the importance of these changes [18–21]. Proteomics approaches are also instrumental in studying interactions between plants and pathogens at the molecular level, providing insights into protein networks and pathways [22]. Thus, the approach holds great promise in improving our understanding of molecular responses in CMV infected cassava plants.

Diverse proteomic approaches have been utilized in the study of the interaction of plants and viral pathogens. Casado-Vela et al. [23] identified protein profiles involved in the defense of tomato plants against *Tobacco mosaic virus* (TMV). This work detected peptidases, endoglucanases, chitinases, proteins associated with the ascorbate–glutathione cycle, pathogenesis-related (PR) proteins, and antioxidant enzymes involved in antiviral plant responses. The comparison of tomato cultivars resistant and susceptible to *Tomato yellow leaf curl virus* (TYLCV) identified 86 differentially expressed proteins and classified them into seven functional groups involved in photosynthesis, protein metabolism, carbohydrate metabolism, energy and amino acid metabolism, detoxification and antioxidation, signal transduction, and chaperone roles. Proteomics analysis has also been used in cassava, to explore responses to abiotic stresses such as drought. However, only two proteomics studies have been performed to study CMV infection. Raghu et al. [24] identified 19 differentially expressed proteins from cassava leaves infected with ICMV and SLCMV using 2D gel electrophoresis and matrix-assisted laser desorption/ionization-time-of-flight (MALDI-TOF) mass spectrometry. Peptide Mass Fingerprint (PMF) analysis indicated that eighteen of these proteins were relevant to plant growth and development and plant defense. A recent report, describing proteome mapping comparing plants susceptible or resistant to South African cassava mosaic virus (SACMV) identified 2,682 differentially expressed proteins during the systemic stage of the infection, and 2,817 differences during the stages of recovery. The key finding of that study was identifying the role of RPL10 protein in NIK1-mediated effector function, triggering plant immunity against the geminivirus infection [25].

Because in Thailand cassava is mostly propagated vegetatively, the use of potentially infected stems during this practice is the primary cause of CMV spread, and farmers also use this approach to replace other cassava cultivars with KU50. Despite previous proteomics studies investigating cassava leaves infected with ICMV and SLCMV, there is no available data on how infected KU50 plants respond to the virus. The objective of this study is to compare the proteome of healthy and infected KU50 leaves to identify changes induced by SLCMV infection. By identifying differentially expressed proteins during the infection, this study contributes significantly to our understanding of molecular mechanisms involved in the pathogenesis of SLCMV infection in cassava.

## Materials and methods

### Sample collection

Healthy and SLCMV infected cassava cv. KU50 stems were vegetatively propagated. The planting materials were supported by which obtained from Thai Tapioca Development Institute (TTDI), Nakhon Ratchasima, Thailand. The stems were cut into 15 cm lengths, containing 3–4 buds each, and planted in 20-cm diameter plastic pots in the green house of the Department of Plant Pathology, Faculty of Kasetsart University, Thailand. After 45 days of cultivation, the apex of healthy and infected stems was collected, pooling leaves from three plants into one sample. To stop all metabolic activity, the leaves were immediately flash frozen in liquid nitrogen, and the frozen samples were stored at -80 °C until protein extraction and nucleic acid extraction.

### CMV detection by PCR

DNA was extracted from the frozen cassava leaves using the CTAB method [26]. Cassava tissues were ground to a powder in liquid nitrogen and 700 µL CTAB buffer was added to 200 mg of ground leaf power. This suspension was incubated at 65 °C for 30 min, then 700 μL chloroform:isoamyl alcohol mixture (24:1) was added to the tubes. DNA was precipitated by the addition of 700 μL isopropanol, and the tubes were incubated at -20 °C for 3 h. The pelleted DNA was washed twice in 70% ethanol and the pellet was dried at room temperature. The DNA was resuspended in ddH_2_O containing 100 μg/ml RNase (Thermo Fisher Scientific, Waltham, MA, USA) and stored at -20 °C. DNA samples were quantitated and checked for integrity by agarose gel electrophoresis and a Nanodrop spectrophotometer (NanoDrop Technologies, Thermo Scientific).

To identify infected plants, the *AV1* gene of SLCMV was amplified using gene specific primers (forward: 5’-GTT GAA GGT ACT TAT TCC C-3’ and reverse: 5’-TAT TAA TAC GGT TGT AAA CGC-3’) [27]. Amplification was performed in a 25-μL reaction volume containing 1 × PCR buffer (PCR Biosystems, London, UK), 0.2 μM each of forward and reverse primers, and 50 ng of the genomic DNA. After an initial denaturation at 94 °C for 5 min 35 cycles were carried out, consisting of denaturation at 94 °C for 40 s, annealing at 55 °C for 40 s, and elongation at 72 °C for 40 s. The final elongation was carried out at 72 °C for 5 min. Amplified products were examined by gel electrophoresis on a 1.5% agarose TAE gel containing RedSafe Nucleic Acid Staining Solution (iNtRON Biotechnology, Sangdaewon, South Korea). Sanger sequencing was used to establish the sequence of the amplified product (Macrogen, The Netherlands), and the identity of the virus was confirmed by BLAST searches in the National Center for Biotechnology Information database (https://blast.ncbi.nlm.nih.gov/Blast.cgi).

### Protein extraction

Cassava tissues were ground to a fine powder in liquid nitrogen, then 1 ml of 0.5% Sodium dodecyl sulfate was added to 100 mg tissue powder. After agitating for 1 h, tubes were centrifuged at 9,000 g for 15 min. The supernatant was transferred to fresh tube, mixed with 1,400 μL of cold acetone, and incubated at -20 °C. The mixture was centrifuged at 9,000 g for 15 min and the supernatant was discarded. The protein pellet was dried and stored at -80 °C.

### Determination of protein concentration using the lowry method

The pellets were resuspended in 0.5% SDS and the protein concentration was determined by the Lowry method [28]. The absorbance at 750 nm (OD_750_) was measured and the protein concentration was calculated using a standard curve established using a serial dilution of BSA.

### In solution digestion

Samples containing 5 µg of total protein were subjected to in-solution protease digestion. Samples were dissolved in 10 mM ammonium bicarbonate (AMBIC) buffer. Disulfide bonds were reduced by adding dithiothreitol (DTT) to a final concentration of 5 mM, followed by an incubation at 60ºC for 1 h. Finally, sulfhydryl groups were alkylated by incubating the samples for 45 min in AMBIC buffer containing 15 mM iodoacetamide, at room temperature, in the dark [29]. Next, samples were mixed with 50 ng/µL of sequencing grade trypsin (1:20 ratio) (Promega, Germany) and incubated at 37ºC overnight, to digest the protein content. Prior to LC–MS/MS analysis, the digested samples were dried and protonated with 0.1% formic acid.

### Protein quantification

Tryptic peptide samples were prepared for injection into an Ultimate3000 Nano/Capillary LC System (Thermo Scientific, UK) coupled to a Hybrid quadrupole Q-Tof impact II™ (Bruker Daltonics) equipped with a Nano-captive spray ion source. Briefly, 1µL of peptide digest was enriched on a µ-Precolumn 300 µm i.d. X 5 mm C18 Pepmap 100, 5 µm, 100 A (Thermo Scientific, UK), separated on a 75-µm I.D. × 15 cm column packed with Acclaim PepMap RSLC C18, 2 μm, 100 Å, nanoViper (Thermo Scientific, UK). The C18 column was enclosed in a thermostatic oven set to 60 °C. Solvent A was 0.1% formic acid in water, solvent B was 0.1% formic acid in 80% acetonitrile. A gradient of 5–55% solvent B was used to elute the peptides at a constant flow rate of 0.30 µL/min over 30 min. Electrospray ionization was carried out at 1.6 kV using CaptiveSpray. Nitrogen was used as drying gas (flow rate approximately 50 L/h). Collision-induced-dissociation (CID) product ion mass spectra were obtained using nitrogen gas as the collision gas. Mass spectra [30] and MS/MS spectra were obtained in the positive-ion mode at *2 Hz* over the range (m/z) 150–2200. The collision energy was adjusted to 10 eV as a function of the *m*/*z* value. The LC–MS analysis of each sample was performed in triplicate. The protein spectral data obtained in this study has been deposited at ProteomeXchange: PXD035792.

### Bioinformatics and data analysis

The MaxQuant software package (version 2.0.3.0) was used to quantitate proteins in individual samples using the Andromeda search engine. This identifies peptide fragments by comparing MS/MS spectra to data deposited in the UniProt *Manihot esculenta* database [31]. Label-free quantitation with MaxQuant standard setting was performed allowing a maximum of two miss cleavages and a mass tolerance of 0.6 Daltons during the main search. These searches were set up based on trypsin being the digesting enzyme, carbamidomethylation of cysteine as a fixed modification, and the oxidation of methionine and acetylation of the protein N-terminus being variable modifications. Only peptides peaks containing a minimum of 7 amino acids and representing a single sequence motif were used in further analysis. Proteins were identified if at least two corresponding peptides were detected, with at least one of these being unique. Only proteins identified based on the above criteria were used for data analysis. False discovery rate (FDR) was set at 1% and was estimated by using reversed search sequences. The maximal number of modifications for any given peptide was set to 5.

For FASTA searches, the proteins present in the *Manihot esculenta* proteome were downloaded from the UniProt database. Potential contaminants, present in the contaminant FASTA file that comes with MaxQuant software, were automatically added to the search space. The MaxQuant ProteinGroups.txt file was loaded into the Perseus search platform (version 1.6.6.0) [31], and potential outliers that did not correspond to any UPS1 protein were removed from the data set. Maximum intensities were log2 transformed and pairwise comparisons were carried out via t-tests. Missing values were imputed in Perseus using a constant value (zero). Data visualization and statistical analyses were conducted using the MultiExperiment Viewer (MeV) of the TM4 software suit [32]. Protein organization and biological function were investigated according to the protein analysis through evolutionary relationships (Panther) protein classification [33] and Venn diagrams were used to visualize differences between protein lists derived from the various samples [34].

### qPCR validation of differentially expressed genes

Total RNA was extracted from healthy and SLCMV infected leaf tissues using QIAzol Lysis Reagent (QIAGEN, Hilden, Germany) according to the manufacturer’s protocol. A RevertAid First Stand cDNA Synthesis Kit (ThermoFisher Scientific, USA) was used for first stand cDNA synthesis, primed by Oligo(dT)_18_, as recommend by the manufacturer. The qPCR reactions were performed using Hot FIREPol® EvaGreen® qPCR Mix Plus (Solis BioDyne,Tartu, Estonia). Reactions contained 10 ng cDNA template, 4 µL 5X HOT FIREPol® EvaGreen® qPCR Mix Plus, 0.2 µM primer mix, and nuclease-free H_2_0 in 20 µL final volume. Amplification was carried out on a CFX Connect Real-Time PCR System (Bio-Rad, USA) using the following cycle parameters: initial denaturation (95ºC for 15 min), followed by 45 cycles of denaturation (95ºC for 30 s), annealing (60ºC for 30 s) and extension (72ºC for 30 s). Fluorescence was recorded at the end of each cycle and a melt curve analysis was carried out between 60ºC-95ºC with a 10 s hold at every 1ºC increment. Each reaction was set up in triplicates. Proteins uniquely upregulated in SLCMV infected cassava samples were analyzed by qPCR experiments. Most of these were associated with gene and protein processing, plant defense, and stress responses. The primers used in these experiments are listed in Supplementary Table S1. The reference gene to assess relative mRNA abundance was *UBQ10* [25]*.* Fold changes in gene expression were analyzed using the log2 ^(ΔCT)^ method [35].

## Results

### SCMV symptoms and virus infection analysis

The characteristic symptoms of SLCMV infection were present in the infected plants after the emergence of the first leaf, with chlorotic mosaics and leaf deformation. PCR experiments using gene specific primers confirmed SLCMV as the causative agent of the visible symptoms. The ~ 900-bp amplicon was only seen when DNA from symptomatic leaves was analyzed (Supplementary Figure S1), and the sequence of the amplified product showed 99% identity with the *Sri Lankan cassava mosaic virus* isolated from Thailand (Supplementary Figure S2).

### Protein identification and quantification

**Fig. 1:**
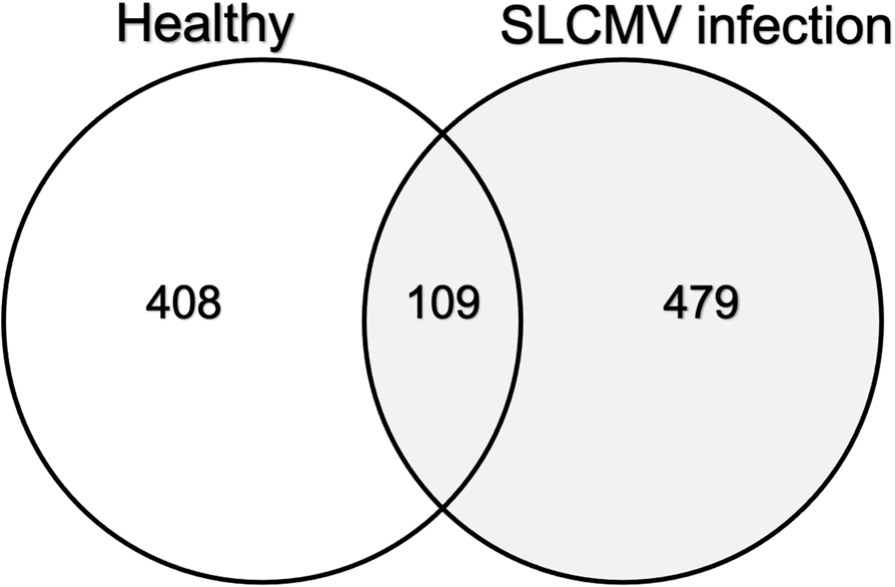
Venn diagram showing the distribution and relationships of proteins in healthy and SLCMV infected KU50 plants

Samples from both healthy and infected plants contained three biotical replicates to reduce random sampling effects and increase the validity of proteins detections. The tandem mass spectra obtained matched the *Manihot esculenta* genome and protein annotation database. After normalizing spectral counts, the protein components of infected samples were compared with healthy leaves to identify differentially expressed molecules. Log_2_ change between paired values was calculated by affinity propagation to identify proteins showing significantly altered expression as a result of the infection. In total, 1,813 peptides were identified, 1,064 in the SLCMV infected KU50 samples and 947 in healthy plants. Venn diagram, showing the relationship between the 479 and 408 proteins detected in SLCMV-infected and healthy cassava plant, respectively is shown in Fig. 1. As shown in the Fig. 1, 109 of these proteins were present in both the infected and healthy samples.

## Gene Ontology Analysis

**Fig. 2:**
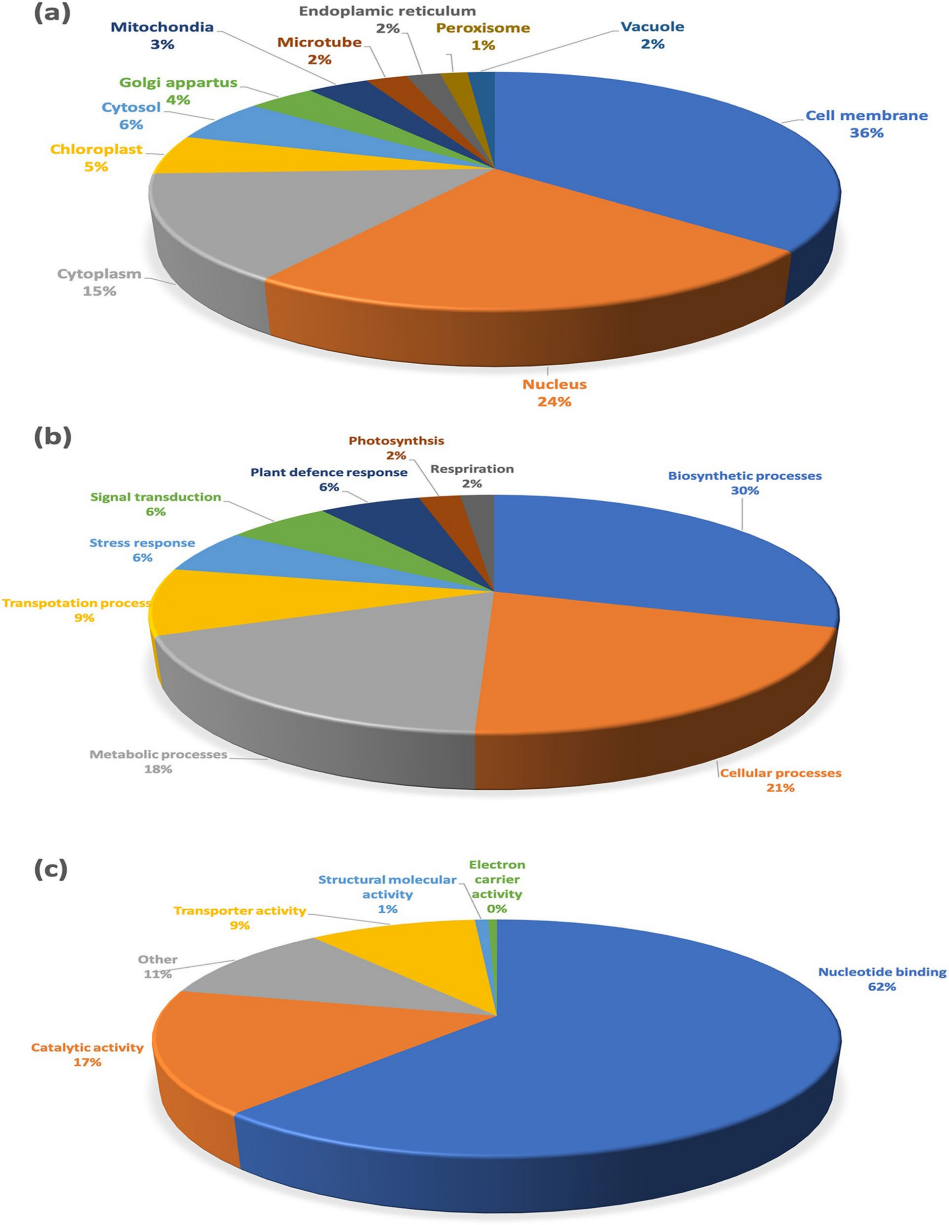
Functional classification of differentially expressed proteins in the leaves of healthy or infected cassava cv. KU50. **A** Proportion of proteins in different functional classes based on cellular localization. **B** Functional grouping of biological processes. **C** Proportion of functional groups based on molecular function

Analyzing the gene ontology classification of the detected proteins showed that 36% of the overexpressed proteins were membrane associated, 23.8% localized in the nucleus, and 14.6% to the cytoplasm. In contrast, proteins in healthy plants were up regulated on membranes (40.1%), nucleus (16.9%) and cytoplasm (19.5%). In addition, both samples contained proteins from other cellular components, such as chloroplast, cytosol, Golgi apparatus, mitochondria, microtube, endoplasmic reticulum, peroxisome, and vacuoles. The cellular localization of the detected molecules is shown in Fig. 2A.

**Fig. 3:**
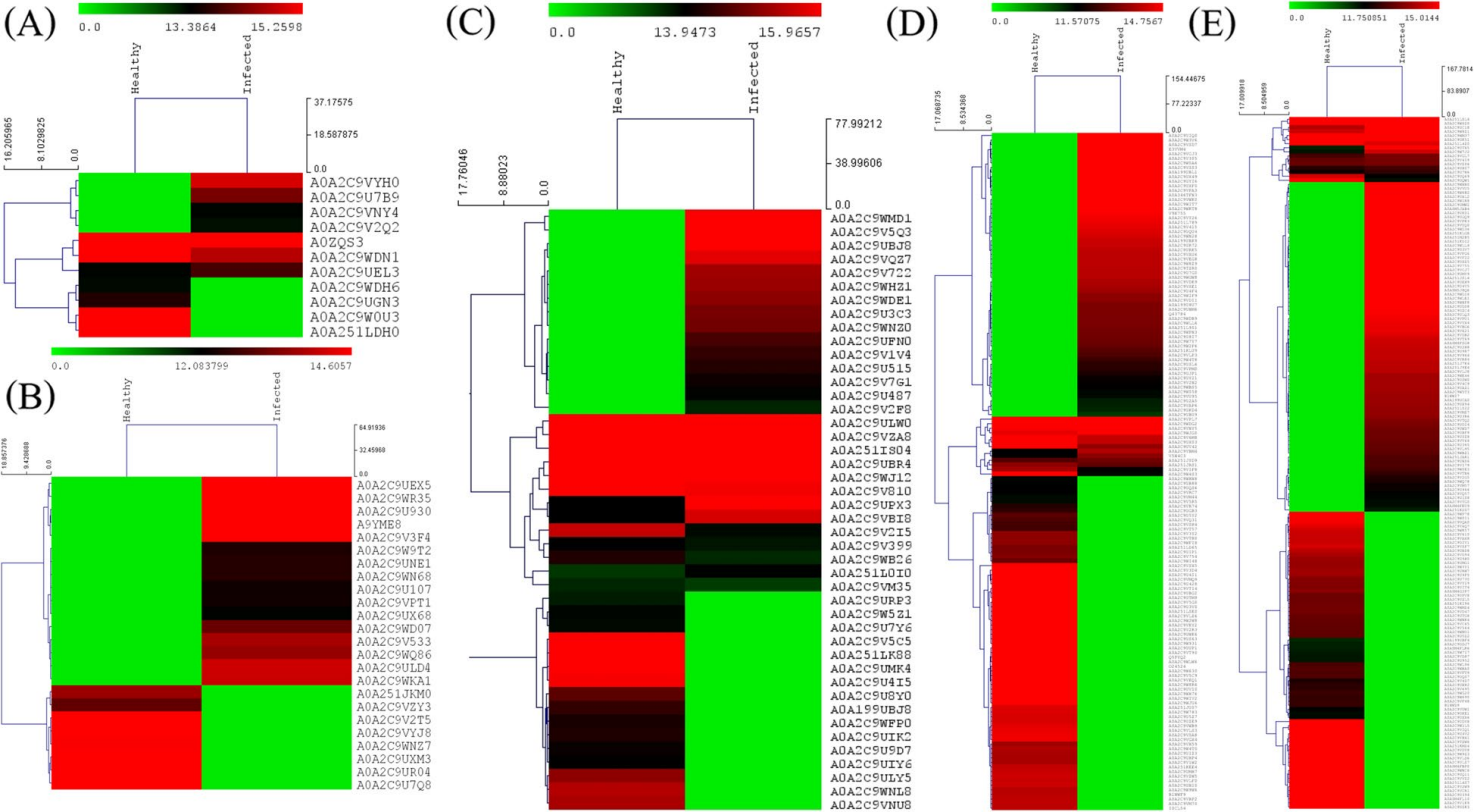
Heatmap of the leaves of cassava cv. KU50 during SLCMV infection. Heatmaps represent each functional group of differentially expressed proteins **a** Respiration; **b** Stress response; **c** Plant defence response; **d** Metabolic processes and **e** Biosynthetic processes

In both healthy and SLCMV-infected cassava plants most of the identified proteins were involved in biosynthetic pathways and cellular or metabolic processes. The proportion of proteins associated with plant defense responses rose from 4.1% in healthy plants to 5.7% as a result of the infection, while the proportion of stress response proteins increased from 3.3% to 5.9%. Proteins related to transportation, signaling, photosynthesis, and respiration are shown in Fig. 2B, while those with a role in nucleotide binding, catalytic activity, transporter activity, structural role, and electron carrier activity are summarized in Fig. 2C. Overall, nucleotide binding proteins represented the most commonly identified protein category both in healthy and infected cells (60%). A heat map of differentially expressed proteins within nine biological functional groups is shown in Fig. 3, where a change in color from red to dark green corresponds to differential changes in gene expression abundance. In general, most upregulated proteins in infected KU50 samples were involved in respiration, plant defense, stress responses, metabolic processes, and biosynthetic pathways (Fig. 3).

## Proteins differentially expressed in response to SLCMV infection

**Fig. 4:**
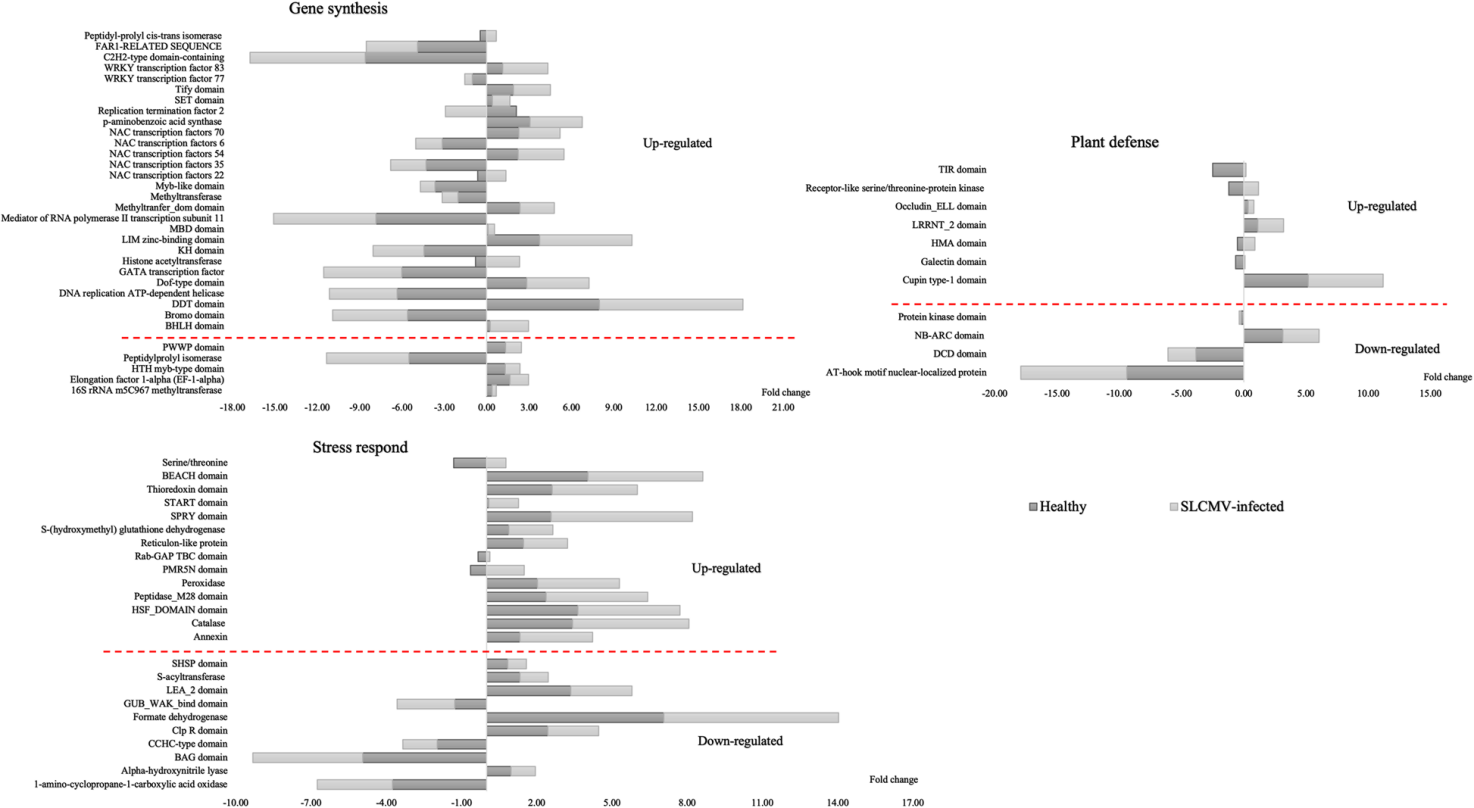
Validation of differential protein expression levels using RT-qPCR. Result represent the averages from three biological replicates and were normalized against the expression level of the endogenous *UBQ10* gene

The identified differentially expressed proteins were associated with biosynthesis, plant defense, and stress responses. Within these categories, a subset of replication, transcription, and translation-related proteins were exclusively expressed in SLCMV-infected samples. There were 59 proteins that were overexpressed more than 17-fold in infected samples. As shown in Table 1, 8 of these were involved in replication, 43 in transcription, and 8 in translation. Interestingly, 21 of the most strikingly upregulated proteins were transcription factors, regulating immune mechanisms, stress responses, and the secretion of plant hormones, while another 20 were connected with plant defense responses. Finally, 26 proteins were previously documented to play a role in biotic plant stress. To confirm the findings of the proteomics analysis, we selected 77 differentially expressed proteins for qPCR-based quantitation. Samples were normalized using the expression of the *UBQ10* gene in the same cDNA sample. These experiments identified 53 mRNAs that were upregulated in SLCMV infected plants (Fig. 4).

**Table 1:** Selected differentially expressed protein of SLCMV infected cassava cv. KU50

No	Majority protein IDs	Protein names	Peptide sequences	Q-value	SLCMV-infected	Healthy
**Gene synthesis**						
1	A0A2C9WNN0	60S acidic ribosomal protein P0	LTKAQKK	0.96771	17.6628	0.00000
2	A0A2C9VDW3	SUZ domain-containing protein	YICSRDYAPSR	0.95952	17.6316	0.00000
3	A0A2C9UVF2	zf-RVT domain-containing protein	EVLSQVFNDRDR	0.96698	17.5503	0.00000
4	A0A2C9VVU5	Histone acetyltransferase	SMHDHVDAWPFKDPVDAR	0.97179	17.5080	0.00000
5	A0A2C9W6B2	Exonuclease domain-containing protein	AAFVTCGNWDVK	0.95586	17.4695	0.00000
6	A0A2C9UHB4	XPGI domain-containing protein	EIDQVQK	0.96768	17.1241	0.00000
7	A0A2C9URT2	C2H2-type domain-containing protein	ICGTREYKCDCGTLFSR	0.97455	17.0298	0.00000
8	A0A2C9W1N8	Protein FAR1	YGAVLCCSSQGFK	0.96147	16.7058	0.00000
9	A0A2C9UMW2	2OG-FeII_Oxy_2 domain-containing protein	SGDVVLMAGEARECFHGKC	0.99538	16.6833	0.00000
10	A0A0M5JAB4	NAC transcription factors 35	EAKYPNGNRSNR	0.95877	16.6756	0.00000
11	A0A2C9UKD1	Replication termination factor 2	ETFSCRSLPLGR	0.96567	16.6197	0.00000
12	A0A2C9UGQ9	MADS-box domain-containing protein	IINKYKK	0.96190	16.4577	0.00000
13	A0A2C9VPK3	BZIP domain-containing protein	ETEDSNHVHGPAQQVKACEMSKF	0.98255	16.3872	0.00000
14	A0A2C9VUQ0	NAC domain-containing protein	QLQDACYDESLWLEHFTSKMK	0.99903	16.3872	0.00000
15	A0A2C9WG36	PUM-HD domain-containing protein	RQEADDLER	1.00600	16.2903	0.00000
16	A0A251KLG8	CBFD_NFYB_HMF domain-containing protein	SNSNSNVREQDR	0.95425	16.1148	0.00000
17	A0A251M2B5	DNA-directed RNA polymerase subunit	DDAGSSAQKSLSESNNLK	0.99818	15.8614	0.00000
18	A0A251KDI2	Peptidyl-prolyl cis–trans isomerase (PPIase)	GNGTGGESIYGMKFADENFK	0.98060	15.8477	0.00000
19	A0A2C9WLL9	PWI domain-containing protein	LASLSPSPER	0.96095	15.8476	0.00000
20	A0A2C9UQH4	Homeobox domain-containing protein	VSDEDEDAINTRK	0.96163	15.7524	0.00000
21	A0A2C9U3V7	ThiF domain-containing protein	AKLLPFK	0.96454	15.6876	0.00000
22	O49169	Elongation factor 1-alpha (EF-1-alpha)	NMITGTSQADCAVLIIDSTTGGFEAGISKDGQTR	0.97401	15.6372	0.00000
23	A0A2C9UXZ5	Eukaryotic translation initiation factor 3 subunit D (eIF3d)	DEEVEARK	0.96404	15.5058	0.00000
24	A0A251IP37	PhoD domain-containing protein	ERTIGPWKNVPR	0.95901	15.50370	0.00000
25	A0A2C9VTP2	MI domain-containing protein	FSEHFTDLPDNFEITSDIAEVFSLSHFVDR	0.82432	15.48880	0.00000
26	A0A2C9VCJ7	Ge1_WD40 domain-containing protein	GEGFSAEK	0.99916	15.40970	0.00000
27	A0A251LEJ6	Histone H4	TLYGFGG	0.95309	15.40470	0.00000
28	A0A2C9V062	AP2/ERF domain-containing protein	WVGEESEVGNSQQQDK	0.97495	15.27920	0.00000
29	A0A2C9UEH9	DNA topoisomerase I	LEDCDFTPIYEWHQR	0.96961	15.24270	0.00000
30	A0A2C9U4V5	AAA domain-containing protein	IYHASVQK	0.96935	15.23960	0.00000
31	A0A0M5J8Q6	NAC transcription factors 22	TTQPLEEK	0.96781	15.23960	0.00000
32	A0A2C9WGI6	Ribonucleoside-diphosphate reductase	IMYNYVDK	0.96702	15.13350	0.00000
33	A0A2C9U749	Glyco_trans_2-like domain-containing protein	EVYAQSISAACQLDWPRDR	0.96982	15.09110	0.00000
34	A0A2C9WLE1	Asparagine–tRNA ligase	LEYLEDR	0.96693	15.06010	0.00000
35	A0A2C9UD08	Methyltransferase	LDLSVMEHYERHCPPPER	0.96683	15.04220	0.00000
36	A0A2C9UST6	BZIP domain-containing protein	EQLLLQV	0.95476	15.03540	0.00000
37	A0A251JD02	MBD domain-containing protein	SEEAEEGKGNVDDDKVQGEDEQPQVEASEGQK	1.06180	14.92410	0.00000
38	A0A2C9VER9	Myb-like domain-containing protein	EAFDARENDRR	0.96093	14.92040	0.00000
39	A0A0M4FSG8	NAC transcription factors 54	QEFSLCRVYKYSK	0.95441	14.34670	0.00000
40	A0A2C9UNL9	BHLH domain-containing protein	EGPNNQLSMFPYLQK	1.01070	14.21040	0.00000
41	A0A251J9E4	Peptidylprolyl isomerase	SILQCSVGHK	0.98228	14.14930	0.00000
42	A0A2C9VLJ8	p-aminobenzoic acid synthase	LAAQLGGAR	0.96271	14.13050	0.00000
43	A0A140H8T7	WRKY transcription factor 83	SEDSGESK	0.99011	13.72130	0.00000
44	A0A2C9UY92	KH domain-containing protein	KTGPEIR	0.96253	13.52750	0.00000
45	A0A2C9U173	SET domain-containing protein	MGQQDDIRESAILEGYR	0.99843	13.44090	0.00000
46	A0A2C9UDI4	16S rRNA m5C967 methyltransferase	YLDHLICSLCHDER	0.96628	13.34490	0.00000
47	A0A140H8T1	WRKY transcription factor 77	WSTSSGR	0.98130	13.32770	0.00000
48	A0A2C9WJL4	HTH myb-type domain-containing protein	PLTQCIWK	0.99094	13.29190	0.00000
49	A0A2C9UBF9	GATA transcription factor	EGADGGTGNGDGRK	1.01230	13.17390	0.00000
50	A0A2C9V4D0	PWWP domain-containing protein	GVSGSTVLESSKETVSDGVASGDSAETR	0.95716	13.09040	0.00000
51	A0A2C9U574	Bromo domain-containing protein	LTFSNAMLYNPPTNYVHKMAESLNK	1.03060	13.07250	0.00000
52	A0A0M4G3M4	NAC transcription factors 6	TNWVMHQYHLGQNEEEKEGELVVSK	1.02060	12.88320	0.00000
53	A0A2C9VJF7	Tify domain-containing protein	FALSCYFHWL	0.96120	12.79210	0.00000
54	A0A251JAR1	DDT domain-containing protein	AAERLQAAQQQAAIER	1.00150	12.77570	0.00000
55	A0A2C9VWW7	LIM zinc-binding domain-containing protein	AHPFWVQKYCPSHEHDGTPR	1.01410	12.70370	0.00000
56	A0A2C9VTN6	DNA replication ATP-dependent helicase/nuclease	YSTPATKK	0.97135	12.43500	0.00000
57	A0A2C9V2G5	Dof-type domain-containing protein	CPRCDSTNTKFCYYNNYSLSQPR	1.00140	12.19830	0.00000
58	A0A2C9U966	Mediator of RNA polymerase II transcription subunit 11	MDPSQTQTSSLQRLQDVEKK	1.01100	11.90330	0.00000
59	A0A0M4FEU9	NAC transcription factors 70	FHPTEEELVGYYLQR	0.97872	11.11290	0.00000
**Plant defense**						
60	A0A2C9V5Q3	TIR domain-containing protein	IMDKFDGHYFVDNVREK	0.97421	17.73090	0.00000
61	A0A2C9W2D3	Occludin_ELL domain-containing protein	FIFSDHDAIQER	0.95675	16.49030	0.00000
62	A0A2C9WG55	DCD domain-containing protein	EFDKIVGCEEDK	0.96774	16.14980	0.00000
63	A0A2C9UBJ8	BEACH domain-containing protein	CYSTNISAVNECQSNGVER	1.00190	16.13340	0.00000
64	A0A2C9VQZ7	Galectin domain-containing protein	FFVALHAR	0.95309	15.76010	0.00000
65	A0A199UBY6	Cupin type-1 domain-containing protein	GKLSWAEDGRELK	0.95784	15.67690	0.00000
66	A0A2C9WEH1	AB hydrolase-1 domain-containing protein	MTRCFSFSEAMNWYHR	0.95826	15.66780	0.00000
67	A0A2C9V722	NB-ARC domain-containing protein	FQYLRMLDLADSK	0.97413	15.22930	0.00000
68	A0A2C9WDE1	Protein kinase domain-containing protein	KYEEIREDWELQYGPQR	0.96563	15.06770	0.00000
69	A0A2C9U3C3	ADP-ribosyl cyclase/cyclic ADP-ribose hydrolase	LNSIDLSHSR	0.95927	14.91910	0.00000
70	A0A2C9WNZ0	zinc_ribbon_12 domain-containing protein	DRAELLR	0.97356	14.86550	0.00000
71	A0A2C9UFN0	Galectin domain-containing protein	ITGEIMRR	0.96827	14.53280	0.00000
72	A0A2C9V1V4	Morc6_S5 domain-containing protein	IVVPMVDYEFNSAKGK	0.98037	14.35230	0.00000
73	A0A2C9V7G1	Receptor-like serine/threonine-protein kinase	CFAQDCK	1.00450	14.07290	0.00000
74	A0A2C9W892	Serine/threonine-protein phosphatase	GAGYTFGQDISEQFNHTNNLK	0.97297	14.01500	0.00000
75	A0A2C9V6E5	AB hydrolase-1 domain-containing protein	VPNLDEAANILLPQTPEK	0.95896	13.66190	0.00000
76	A0A2C9VHG8	LRRNT_2 domain-containing protein	PAWFVMMVEGYQKSR	0.98067	13.58790	0.00000
77	A0A2C9ULD8	HMA domain-containing protein	MTGTDSVSVNVLEK	0.98116	13.52200	0.00000
78	A0A2C9VG49	AT-hook motif nuclear-localized protein	KPDLGISMSNSNR	0.97555	13.43680	0.00000
79	A0A2C9V2F8	C2 domain-containing protein	LSTKSDDSGSTR	1.01350	11.97780	0.00000
**Stress responds**						
80	A0A2C9WMD1	Catalase	SLLEDEAIR	1.00450	18.67530	0.00000
81	A0A2C9VL88	PMR5N domain-containing protein	DDLTRPLYDESECPYIQPQLTCQEHGR	0.95505	17.18910	0.00000
82	A0A251KQD0	Rab-GAP TBC domain-containing protein	EQEAAMLQVLMR	0.96984	17.18190	0.00000
83	O49893	Alpha-hydroxynitrile lyase	VYQVQGGDHK	0.98293	17.11850	0.00000
84	A0A2C9V3F4	Peptidase_M28 domain-containing protein	GISQWAHGFK	0.98118	16.73150	0.00000
85	A0A2C9U930	Formate dehydrogenase, mitochondrial (FDH)	EWLESKGHRYIVTDDK	0.96286	16.17200	0.00000
86	A0A2C9UBJ8	BEACH domain-containing protein	CYSTNISAVNECQSNGVER	1.00190	16.13340	0.00000
87	A0A2C9WR35	S-acyltransferase	PMVQQDSLK	0.96191	15.59860	0.00000
88	A0A2C9VMF9	Clp R domain-containing protein	AWLRDFFDQVDR	0.96744	15.41910	0.00000
89	A0A2C9UMY9	BAG domain-containing protein	EVDEIEKRISMK	0.95848	15.40440	0.00000
90	A0A2C9WIP6	LEA_2 domain-containing protein	ATTHAPPSR	0.96128	15.06010	0.00000
91	A0A2C9UEX5	Peroxidase	EALGNNNSAR	0.96044	14.73540	0.00000
92	A0A2C9UKE3	GUB_WAK_bind domain-containing protein	ACEATGGTCGYGTDGIR	0.98126	14.65070	0.00000
93	A0A2C9UUL9	CCHC-type domain-containing protein	ELFSHFENLNPDQLMVTEQKR	0.40909	14.45930	0.00000
94	A0A2C9WKA1	START domain-containing protein	RIWELDGSYYCVTK	0.96435	14.09110	0.00000
96	A0A2C9V533	SHSP domain-containing protein	QFRLPNNADLDHIK	0.97616	13.76390	0.00000
97	A0SVL8	1-amino-cyclopropane-1-carboxylic acid oxidase	EPRFEAMKAVENNVNLGPNCYCLIINYY	1.06270	12.79130	0.00000
98	A0A2C9WFP1	Thioredoxin domain-containing protein	SYDAARSSTSSSSLGQITLSQTNEAK	1.03710	12.77430	0.00000
99	A0A2C9WN68	HSF_DOMAIN domain-containing protein	HNNFSSFVRQLNTYGFK	0.70270	12.46310	0.00000
100	A0A2C9UNE1	S-(hydroxymethyl)glutathione dehydrogenase	VRAATGVGIMMNDRK	0.97770	12.45550	0.00000
101	A0A2C9VPT1	Reticulon-like protein	NLFENFNKR	0.97989	12.26180	0.00000
102	A0A2C9U107	PH domain-containing protein	RNKPPQFPTR	0.99184	12.24640	0.00000
103	A0A2C9UX68	Annexin	ETSGDYK	0.97739	11.92120	0.00000
106	A0A2C9U854	SPRY domain-containing protein	IAVVVFPKPEELK	0.95840	10.87570	0.00000
107	A0A2C9UGF4	HVA22-like protein	EASGAFR	0.98123	9.993220	0.00000

A total of 29 highly upregulated transcripts were related to biosynthetic processes, including the transcription factors NAC 6, NAC 22, NAC 35, NAC 54, NAC 70, WRKY 77, and WRKY 83. Furthermore, mRNA abundance and protein expression levels showed good correlation for these molecules. Regarding genes that were involved in transcription, translation, and protein synthesis DNA replication ATP-dependent helicase (A0A2C9VTN6) and replication termination factor 2 (A0A2C9UKD1) were also upregulated at transcript levels, showing good correlation with the proteomics data. However, transcripts levels for 16S rRNA m5C967 methyltransferase (A0A2C9UDI4), elongation factor 1-alpha (EF-1-alpha, O49169), HTH myb-type domain-containing protein (A0A2C9WJL4), peptidylprolyl isomerase (A0A251J9E4), and PWWP domain-containing protein (A0A2C9V4D0) were not upregulated at mRNA level, indicating discrepancies between proteomics and transcription data (Fig. 4). Furthermore, proteomics data showed that the WRKY 18 (A0A140H8M2), WRKY 24 (A0A140H8M8) and WRKY 29 (A0A140H8N3) transcription factors were down regulated in SLCMV infection.

qPCR experiments also confirmed changes in the abundance of mRNAs encoding plant defense molecules. The seven upregulated transcripts were cupin type-1 domain (A0A199UBY6), galectin domain (A0A2C9VQZ7), heavy metal-associated domain (HMA domain) (A0A2C9ULD8), Leucine rich repeat N-terminal domain (LRRNT_2 domain) (A0A2C9VHG8), occludin_ELL domain (A0A2C9W2D3), receptor-like serine/threonine-protein kinase (A0A2C9V7G1), and Toll/interleukin-1 receptor/resistance protein domain (TIR domain) (A0A2C9V5Q3). At the same time, AT-hook motif nuclear-localized protein, DCD domain, NB-ARC domain and, protein kinase domain were down-regulated according to qPCR and proteomics analysis (Fig. 4).

The expression of an additional 24 molecules was determined at mRNA level. Fourteen of these, annexin (A0A2C9UX68), catalase (A0A2C9WMD1), HSF_DOMAIN domain (A0A2C9WN68), peptidase_M28 domain (A0A2C9V3F4), peroxidase (A0A2C9UEX5), PMR5N domain (A0A2C9VL88), Rab-GAP TBC domain (A0A251KQD0), reticulon-like protein (A0A2C9VPT1), S-(hydroxymethyl) glutathione dehydrogenase (A0A2C9UNE1), SPRY domain (A0A2C9U854), START domain (A0A2C9WKA1), Thioredoxin domain (A0A2C9WFP1), and BEACH domain (A0A2C9UBJ8) showed elevate transcript abundance, in concordance with LC–MS/MS analysis. However, transcript levels of 1-amino-cyclopropane-1-carboxylic acid oxidase (A0SVL8), alpha-hydroxynitrile lyase (O49893), BAG domain (A0A2C9UMY9), CCHC-type domain (A0A2C9UUL9), Clp R domain (A0A2C9VMF9), formate dehydrogenase (A0A2C9U930), GUB_WAK_binding domain (A0A2C9UKE3), LEA_2 domain (A0A2C9WIP6), S-acyltransferase (A0A2C9WR35), and SHSP domain (A0A2C9V533) were found to be downregulated during qPCR, seemingly contradicting proteomics findings (Fig. 4).

## Discussion

We explored the consequences of SLCMV infections in cassava plants at the level of the pant proteome. This approach enabled the documentation of changing protein expression levels in response to the infection and provided valuable insights into the molecular processes that occur in infected plants. Apart from their relevance to basic biological sciences, the findings may provide crucial information to aid the development of SLCMV tolerant commercial cultivars. Developing such resistant plants is critical for disease control and has very high economic significance. This is the first documentation of global changes in the cassava proteome, comparing healthy and SLCMV infected KU50 plants. Subsequent gene ontology analyses highlighted the most likely biological processes altered by this viral infection. During the analysis we paid special attention to proteins that have been implicated in geminivirus infestations in other plants, particularly those molecules involved in gene synthesis, and plant defense and stress responses.

The expression of an extensive subset of differentially expressed proteins was also quantitated using qPCR, including a set of 77 proteins upregulated during SLCMV infection. Out of this fairly comprehensive set of molecules, qPCR confirmed a corresponding increase in mRNA abundance in approximately 70% of the cases. However, proteomics findings and qPCR gene expression data showed contradictory outcomes in the remaining 30%. While this discordance may seem striking, several mechanisms have been reported in the literature that may explain such discrepancies. Post-transcriptional regulatory processes, such as different local translation efficiency and codon bias [36], altered translation elongation rate [37], differences in the half-life of proteins [38], or variations in poly A tail length [39, 40] have all been reported to result in discordant proteomics/transcriptomics results.

According to gene ontology analysis, the majority of differentially expressed proteins in SLCMV infected cassava cv. KU50 plants localized to the cell membrane, nucleus, cytoplasm, and chloroplast. Previous studies indicated that plant defense pathways involved transcriptional and post-transcriptional gene silencing, the ubiquitin-proteasomal degradation pathway, protein kinase signaling cascades, autophagy, and hypersensitive responses [41]. In the nucleus, geminivirus use the enzymatic machinery of the hosts to replicate their initially ssDNA genomes to double stranded forms that interact with histones in the form of minichromosomes. This elicits RNA-directed DNA methylation (RdDM) to suppress the virus via transcriptional gene silencing (TGS) [42–44]. Beyond their role in photosynthesis, chloroplasts also play an important role in plant immunity through the regulation of reactive oxygen species (ROS) and salicylic acid (SA) signaling. Chloroplasts also modulate biosynthetic pathways via the synthesis of phytophomones, gibberellic acid (GA), abscisic acid (ABA) and jasmonic acid (JA). During the infection, chloroplasts appear to be one of the major targets of pathogens [45, 46]. The communication between the chloroplast and the nucleus has been explored during biotic stresses. This process, pattern-triggered immunity (PTI) in thylakoid membrane, involves calcium sensing receptors (CAS) inducing the reprogramming of transcriptional activity, SA synthesis, and callose deposition, leading to pathogen resistance [47, 48]. In addition, nuclear transcription factors [45] and several cell surface membrane proteins, including receptor serine threonine kinase (RSTK), Toll/interleukin-1 receptor (TIR), and leucine rich repeat N-terminal domain (LRRNT 2) protein cascades, were reported to play a critical role in plant immunity [49].

Transcription factors (TFs) are keys regulators of mRNA synthesis, acting on *cis*-regulatory elements in promoter regions. Recent work indicated that TFs in the WRKY, AP2/ERF (Apetala2/Ethylene Responsive Factor), NAC (NAM, ATAF and CUC), MYB, bZIP (Basic leucine zipper domain), bHLH (Basic helix-loop-helix), NF-Y (Nuclear Factor Y), and CAMTA (CaM-binding transcription activator) TF families play a central role in regulating plant immune responses [50, 51]. We detected the upregulation of WRKY 77 and WRKY 83 in SLCMV infected cassava plants. Wei et al. [52] identified a group of *Manihot esculenta* (Me)WRKY transcription factors, which were shown to be involved in innate immunity, plant defense mechanisms, and biotic stress responses [53]. In agreement with our own observations, Freeborough et al. [54] showed that *MeWRKY 83* was up regulated during the late stages of SLCMV infection in a susceptible cassava cultivar (T200). In our experiments, we also detected the overproduction of *MeWRKY 83* both at protein and transcript level. Interestingly, a novel cluster of genes containing WRKY TF binding sequences was identified in some plants [55]. Because these genes appear to have a role in plant responses during infections, there is an urgent need to explore WRKY binding domain-containing genes in cassava, as these may aid the identification of CMD resistant variants.

We also detected the upregulation of NAC 6, NAC 35, NAC 22, NAC 54, and NAC 70 transcription factors in SLCMV infected leaves, while in healthy plants *NAC* genes were expressed at a low background levels. Using high throughput RNA sequencing, a previous study, comparing cassava brown streak virus sensitive and resistant plant variants also detected the increased expression of the members of the NAC family of TFs in resistant cultivars [56]. These TFs play a crucial role in responses to pathogens and abiotic stressors [57]. NAC 22 and NAC 35 were up regulated in response to salt, cold, and osmotic stress, or after treatment with ABA or H_2_O_2_ [58]. Our experiments suggest that NAC 54 and NAC 70 are upregulated in a more specific way, after an SLCMV infection. However, the exact role of NAC 54 and NAC 70 during a viral infection is currently unclear and will require further studies.

MYB TFs were also overexpressed during SLCMV infection. In *Arabidopsis,* the myeloblastosis response to pathogen attacks occurs via the activation of a hypersensitive cell death program, through the brassinosteroid pathway [59]. MYB plays an important role in signal transduction after exposure to SA [60], ABA [61], GA [62], and JA [63]. Recently, Wang et al. [64] reported that MeMYB26 was associated with drought resistance traits and biomass storage. Furthermore, the MeMYB pathway may show a crosstalk with MeWRKYs TFs under condition of stress in cassava [65]. As reported by Ramulifho and Rey [25] ribosomal protein L10 (RPL10) plays role in protecting cassava against geminivirus infections, with a MYB domain-containing TF activating RPL10 transcription in the nucleus. This mechanism inhibited gene translation by the host and had a profound inhibitory effect on geminiviral replication. The authors proposed that the MYB/RPL10 protein network contributed to the symptomatic recovery of the virus resistant TIM3 cassava cultivar after exposure to South African cassava mosaic virus infection. However, while the abundance of MYB protein also showed an increase in response to SLCMV in our studies, we were unable to detect any RPL10. This may indicate that MYB-driven responses may vary according to the strain of geminivirus causing the infection, or the cultivar being tested. Nonetheless, determining MYB and RPL10 expression could be a valuable tool in breeding of CMD resistant cultivars. The fact that RPL10 is encoded on chromosome 12 of cassava and that polymorphisms in this chromosome appear to correlate with CMD2 resistance [66, 67], lends further support to the potential importance of the MYB/RPL10 pathway.

Basic helix-loop-helix (bHLH) proteins are transcription factors representing one of the largest TF families [68]. In *Arabidopsis*, bHLHs can act both as transcriptional activators or repressors, influencing plant development and physiological processes, such as phytohormone [69] and secondary metabolite synthesis [70, 71]. JA regulates plant stress responses after pathogen attacks and wounding [72, 73]. Wang et al. [74] identified 96 bHLH genes in tomato plans that were either resistant or susceptible to yellow leaf cure virus (TYLCV). In our proteomics data, bHLH was overexpressed and the gene expression was also upregulated by a factor of 2 following SLCMV infection (Table 1 and Fig. 4). However, JA responses after SLCMV infestation or other viral infections will require further studies.

The 60S acidic ribosomal protein P0 (A0A2C9WNN0), 50S ribosomal protein L16 (B1NWI7), ribosomal_S7 domain-containing protein (A0A2C9V9X4), and ribosome biogenesis protein NOP53 (A0A2C9VL95) were also overexpressed in SLCMV infected KU50 (Table 1 and Supplementary Table S1). Several studies have reported the direct interaction of ribosomal proteins with plant viruses during infection. Such interactions were noted in infections caused by *Soybean mosaic virus* (SMV), *Sugarcane mosaic virus* (SCMV), *Rice yellow mottle virus* (RYMV), *Papaya meleira virus* (PMeV), *Pepper mild mottle virus* (PMMoV), Rice black-streaked dwarf virus (RBSDV), BNYVV, and *Mungbean yellow mosaic India virus* (MYMIV) [75, 76]. In addition, our experiments also showed the upregulation of elongation factor 1-alpha (EF-1-alpha) (O49169) and eukaryotic translation initiation factor 3 subunit D (eIF3d) (A0A2C9UXZ5) in response to SLCMV infection (Table 1). Transcription initiation and elongation factors interact with viral particles during viral protein synthesis, and have been shown to be differentially expressed during SCMV, RYMV, PMeV, RBSDV, and MYMIV infections [75–80]. Cho et al. [81] investigated how elongation factors and ribosomal proteins interacted with PVX stem loop 1 RNAs. Interestingly, in a resistant rice cultivar, translation elongation factor and RMYV co-purified, indicating particularly strong interactions [81]. Protein synthesis during viral replication and the interactions between viral nucleic acids and host proteins represent a unique field in biology. Our proteomics data provide an early insight into how elongation factor/ ribosomal protein interactions may influence SLCMV infections.

Our experimental data indicated the differential regulation of pathogen defense and stress response proteins, representing 5.9 and 5.7%, of all identified proteins, respectively (Fig. 2). Plants receptor serine threonine kinases (RSTK), Toll/interleukin-1 receptor (TIR), and leucine rich repeat N-terminal domain (LRRNT_2) were significantly upregulated in SLCMV infected plants, confirming the findings of previous studies [82]. The nucleotide-binding site leucine-rich repeat (NBS-LRR) family of receptors, for example receptor serine threonine kinase (RSTK) proteins, are involved in disease resistance and the regulation of plant development, as well as self-versus non-self-discrimination. RSTK is a transmembrane protein that, in associations with other protein families, regulates both disease resistance and developmental processes [83]. Plants respond to pathogen-associated molecular pattern molecules (PAMPs), using extracellular leucine-rich repeat (LRR) receptors for their recognition, serine/threonine kinases (STKs) for down-stream processing, and mitogen-activated protein kinases (MAPKs) for signal transduction. Pattern recognition receptors (PRRs) are activated upon detecting predetermined molecular structures, leading to the initiation of PAMP-triggered immunity (PTI) that can prevent the entry of most invading organisms [84]. Recent research has shown that viruses can both stimulate and repress PTI-like mechanisms [85–99]. In some instances, pathogen-derived molecular structures suppress protective mechanisms. This phenomenon is referred to as effector-triggered susceptibility (ETS) [86, 87]. To counteract ETS, a set of intracellular nucleotide-binding leucine-rich repeat (NLR) receptors have evolved, providing effector-triggered immunity [88]. These recognise virulence factors produced by the pathogen in a very precise manner and activate a second phase of defense responses [89].

**Fig. 5:**
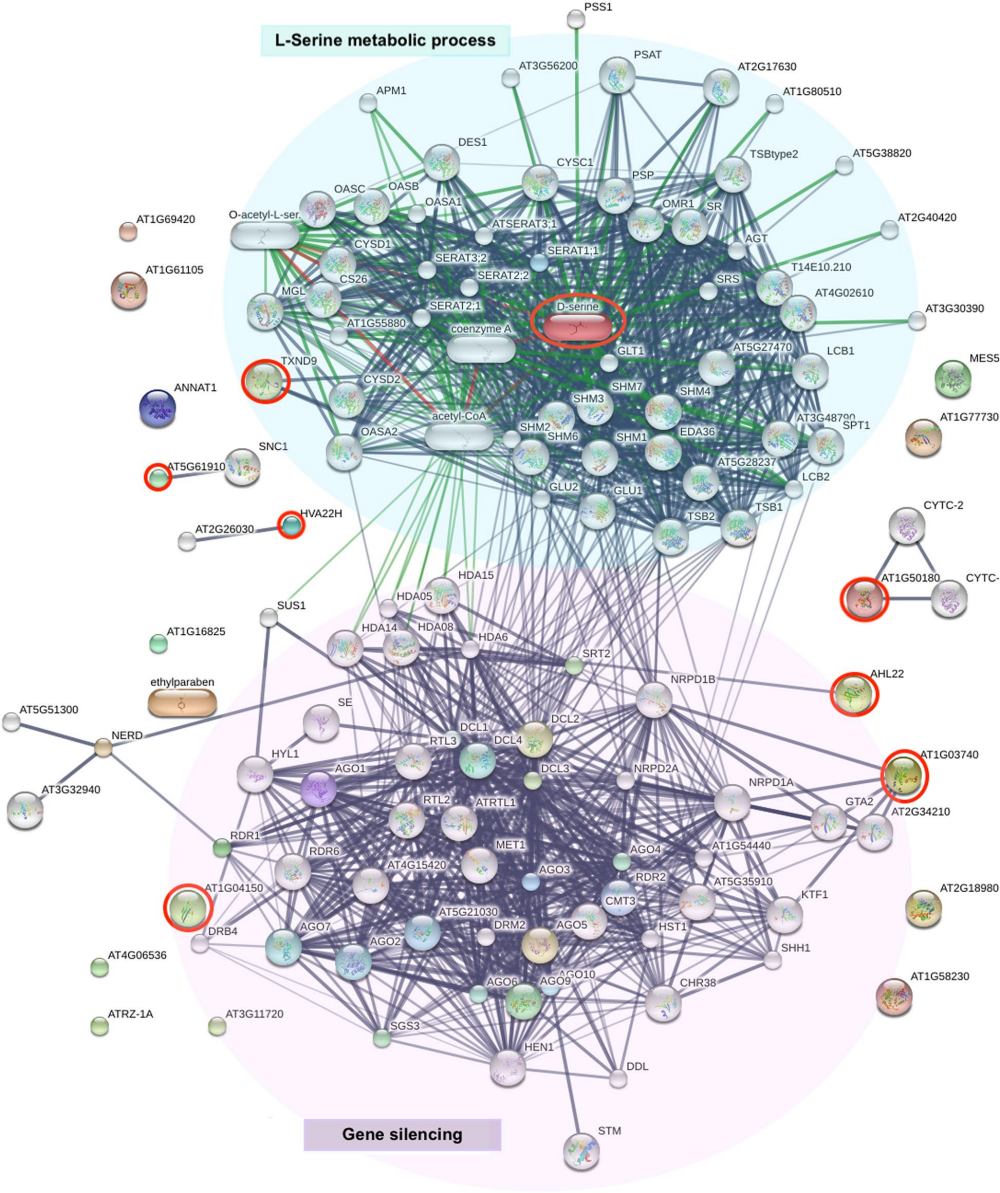
Prediction of protein induced gene silencing and L-serine metabolic process using STICH analysis

The Search Tool for Interacting Chemicals (STITCH) algorithm was developed to investigate interactions between proteins and small molecules during biological processes [90]. In our work, we noted that the AT-hook motif nuclear-localized protein (AHL22) directly interacted with histone deacetylase 6 (HDA6), initiating a repression with a significant role in transcriptional regulation (Fig. 5). Previous work indicated that the *AHL* gene family is necessary for plant growth, development, and stress responses. In particular, AHL22 suppresses hypocotyl growth in Arabidopsis [91]. Taken together, this information suggests that the nuclear localization of an AT-hook motif protein in SLCMV infected cassava may indicate gene silencing. Protein kinase domain-containing protein (AT1G03740) is also involved in gene silencing though its interactions with RNA polymerase (NRPD1B), transcription elongation factor (AT2G34210), and the transcription factors KTF1 and GTA2 TFs (Fig. 5). Protein kinases represent one of the largest protein families in plants. Their central role in environmental stress responses, such as drought, high salinity, cold, and pathogen attacks has been amply demonstrated. In *Arabidopsis*, the serine/threonine protein kinase, TOUSLED (TSL) is required for antiviral RNA silencing and regulates RNA interference [92]. Wang et al. [93] identified the putative substrates of nine protein kinases that function in plant abiotic and biotic stress responses. This work contains more than 5,000 putative target sites controlling cellular activities. It is hoped that our results will represent the starting point for further studies on the mechanisms underlying plant responses to environmental stresses, including the mechanisms that involve gene silencing.

Serine/threonine-protein phosphatases use D-serine while thioredoxin domain-containing protein (TXND9) interacts with L-serine during metabolic processes. Serine metabolism in plant has been studied in the context of photorespiration, where serine is formed from two molecules of glycine. This serine becomes a precursor for glycine, formate, and glycolate accumulation during conditions of stress. Serin/threonine protein phosphatases, especially PP2A, fulfill multiple roles in plant cells during growth and stress rated signaling. Hormone-related signal transduction and oxidative stress signaling regulate the activity of a range of enzymes involved in key metabolic pathways [94]. proposed that this signaling pathway was essential in connecting carbon and nitrogen metabolism, preserving the cellular redox potential, and energy levels in plants under stress. Exposure to exogenous H_2_O_2_ replicates some of the consequences of *Rhizoctonia cerealis* infection by increasing the expression of PP2Ac [96]. There have been numerous studies showing the conflicting roles of Thioredoxins (TRXs) in viral pathogenesis and resistance. In *Nicotiana tabacum*, TRXh3 confers resistance to Tobacco mosaic virus (TMV) and Cucumber mosaic virus (CMV) [97] and in maize TRX mediates early resistance against the *Sugarcane mosaic virus* [98]. Using yeast-two-hybrid screening combined with invitro pull-down and biomolecular fluorescent complementation (BiFC) assays, Mathioudakis et al. [99] reported that thioredoxin (TRX) in tomato plants interacted with the pepino mosaic virus (PepMV) coat protein, triple gene block protein (TGBp1), heat shock cognate protein 70, and catalase 1 (CAT1). In our study, STICH analysis predicted the interaction of TRX combined with glutamate synthetase 1 and 2 (GLT1 and 2), potentially affecting glutamate biosynthesis.

Identifying viral and host components associated with infections and understanding the molecular mechanisms of plant defense systems, pathogen virulence, and susceptibility, are key areas of research in plant virology. The results presented here provide an overview of some of the key protein level changes induced by SLCMV infections in cassava plants. A better understanding of the fundamental processes involved in viral infections and host responses is critical for developing and selecting more resistant crops. An ideal defense mechanism would prevent the replication of the virus altogether, eliminating any chance of viral evolution. Additionally, a combinational or additive strategy, targeting the virus and its vector simultaneously, would hinder the infection even more, offering crops more durable protection.

## Conclusions

Here we describe the comprehensive proteomics analysis of the leaves of SLCMV infected and healthy cassava KU50 cultivar plants. In the course of the work carried out, more than 1,813 proteins were detected, including 479 that were upregulated in infected plants. These SLCMV induced proteins could be classified into nine functional groups and exhibited a wide range of biological functions. Further studies of the differentially regulated molecules in the cell membrane, nucleus, and chloroplast will help the identification of key proteins involved in cassava-SLCMV infection, potentially aiding the development of resistant crops.

## Supplementary Information

**Additional file 1.**

**Additional file 2.**

**Additional file 3.**

**Additional file 4.**

## Data Availability

The datasets generated and analyzed in the current study are available in ProteomeXchange; accession number PXD035792 and the data sets supporting the conclusions of this article are included with the article and its additional files.
