# Supplementary material for: Analysis of proteomic changes in cassava cv. Kasetsart 50 caused by Sri Lankan cassava mosaic virus infection
Source: BMC Plant Biol. 2022 Dec 10;22:573. doi: 10.1186/s12870-022-03967-1 (PMC9737768; doi:10.1186/s12870-022-03967-1)
Supplement: Supplementary file 1 — Additional file 1. [file 12870_2022_3967_MOESM1_ESM.pdf]

~900 bp

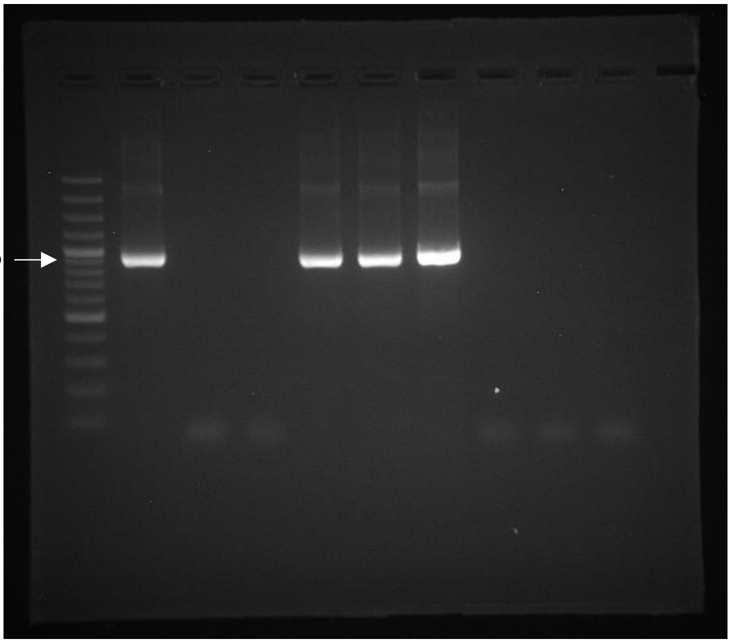

Figure S1. Detection of SLCMV in healthy and infected cassava cv. KU50 leaves, using PCR. Lane 1: SLCMV infected sample, Lane 2: non-infected sample, Lane 3: NTC, Lanes 4-6: amplicons from SLCMV pathogenicity tests, and Lanes 7-9: non infected sample from the same experiments.
