# Supplementary material for: Analysis of proteomic changes in cassava cv. Kasetsart 50 caused by Sri Lankan cassava mosaic virus infection
Source: BMC Plant Biol. 2022 Dec 10;22:573. doi: 10.1186/s12870-022-03967-1 (PMC9737768; doi:10.1186/s12870-022-03967-1)
Supplement: Supplementary file 2 — Additional file 2. [file 12870_2022_3967_MOESM2_ESM.pdf]

Figure S2. Partial *AV1* gene nucleotide sequence of SLCMV

```
tcgaagcgac cagcagatat catcatttca actcccgcct cgaaggttcg tcgccgtctg aacttcgaca
gcccatacag cagtcgtgct gctgtcccca ttgtccgcgt cacaaaaaga caagcctgga caaacaggcc
catgaatcgg aagcccaggt ggtacaggat gttcaaaagc ccagatgttc ctaggggatg tgaaggccca
tgtaaggttc aatcgtttga gtccagacac gatgtggtcc atataggtaa ggtcatgtgc atctctgatg
tcactcgtgg agttgggctt actcatcgcg tgggtaagag gttttgcgtt aagtccgttt atatcctggg
taagatatgg atggatgaaa atattaagac caagaatcat acgaatagtg tgatgttctt ccttgtaagg
gatcgtaggc ctgttgataa gccccaggat tttggtgaag tgtttaatat gttcgataat gaacctagta
cagctacggt gaagaacatg catcgtgatc gttatcaagt cctcaggaag tggagtgcca ctgtcactgg
tggtcagtat gcgagcaagg aacaggcttt agttaggcgt ttttttagag ttaataatta tgttgtgtat
aaccagcaag aggctggcaa gtatgaaaat cataccgaga atgcattgat gctgtacatg gcgtgtactc
atgcctctaa ccctgtatac gctacgctga agattagaat ctacttctac gattcggtca gcaattaata
aacattaaat tgtattaaat tagactgctc aatactgtca gtcccagcga
```

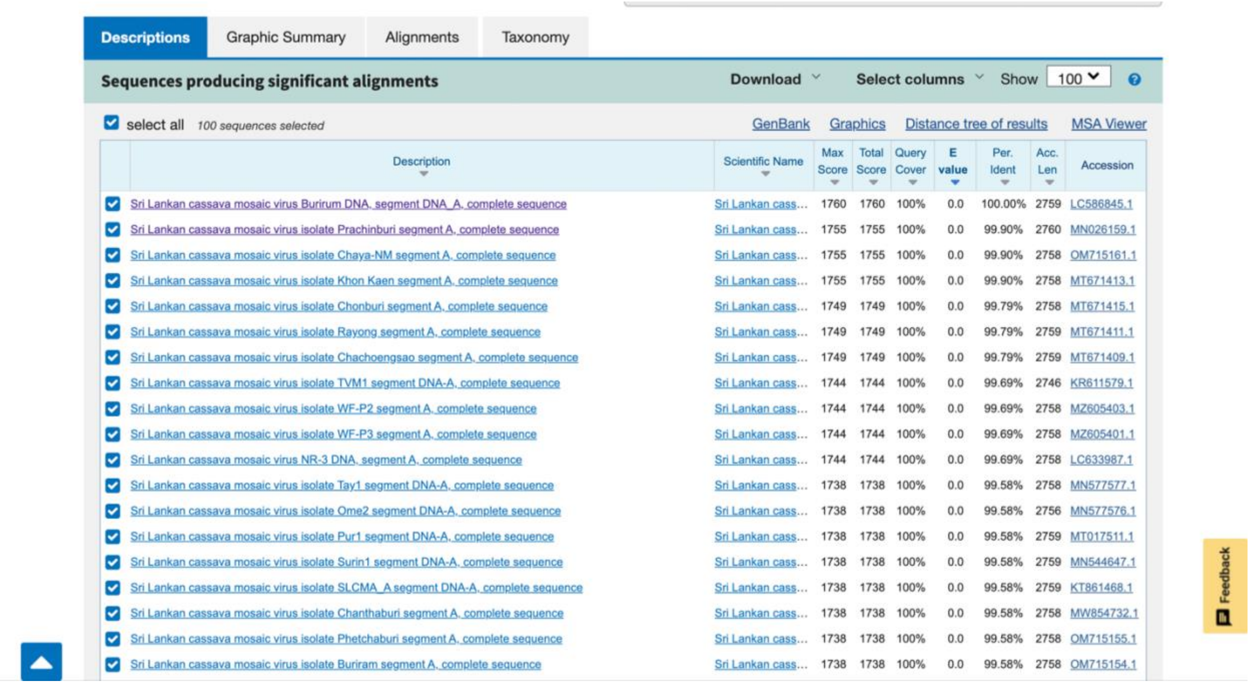
Descriptions | Graphic Summary | Alignments | Taxonomy

Sequences producing significant alignments — Download — Select columns — Show 100

select all *100 sequences selected* — GenBank — Graphics — Distance tree of results — MSA Viewer

Description	Scientific Name	Max Score	Total Score	Query Cover	E value	Per. Ident	Acc. Len	Accession
Sri Lankan cassava mosaic virus Burirum DNA, segment DNA_A, complete sequence	Sri Lankan cass...	1760	1760	100%	0.0	100.00%	2759	LC586845.1
Sri Lankan cassava mosaic virus isolate Prachinburi segment A, complete sequence	Sri Lankan cass...	1755	1755	100%	0.0	99.90%	2760	MN026159.1
Sri Lankan cassava mosaic virus isolate Chaya-NM segment A, complete sequence	Sri Lankan cass...	1755	1755	100%	0.0	99.90%	2758	OM715161.1
Sri Lankan cassava mosaic virus isolate Khon Kaen segment A, complete sequence	Sri Lankan cass...	1755	1755	100%	0.0	99.90%	2758	MT671413.1
Sri Lankan cassava mosaic virus isolate Chonburi segment A, complete sequence	Sri Lankan cass...	1749	1749	100%	0.0	99.79%	2758	MT671415.1
Sri Lankan cassava mosaic virus isolate Rayong segment A, complete sequence	Sri Lankan cass...	1749	1749	100%	0.0	99.79%	2759	MT671411.1
Sri Lankan cassava mosaic virus isolate Chachoengsao segment A, complete sequence	Sri Lankan cass...	1749	1749	100%	0.0	99.79%	2759	MT671409.1
Sri Lankan cassava mosaic virus isolate TVM1 segment DNA-A, complete sequence	Sri Lankan cass...	1744	1744	100%	0.0	99.69%	2746	KR611579.1
Sri Lankan cassava mosaic virus isolate WF-P2 segment A, complete sequence	Sri Lankan cass...	1744	1744	100%	0.0	99.69%	2758	MZ605403.1
Sri Lankan cassava mosaic virus isolate WF-P3 segment A, complete sequence	Sri Lankan cass...	1744	1744	100%	0.0	99.69%	2758	MZ605401.1
Sri Lankan cassava mosaic virus NR-3 DNA, segment A, complete sequence	Sri Lankan cass...	1744	1744	100%	0.0	99.69%	2758	LC633987.1
Sri Lankan cassava mosaic virus isolate Tay1 segment DNA-A, complete sequence	Sri Lankan cass...	1738	1738	100%	0.0	99.58%	2758	MN577577.1
Sri Lankan cassava mosaic virus isolate Ome2 segment DNA-A, complete sequence	Sri Lankan cass...	1738	1738	100%	0.0	99.58%	2756	MN577576.1
Sri Lankan cassava mosaic virus isolate Pur1 segment DNA-A, complete sequence	Sri Lankan cass...	1738	1738	100%	0.0	99.58%	2759	MT017511.1
Sri Lankan cassava mosaic virus isolate Surin1 segment DNA-A, complete sequence	Sri Lankan cass...	1738	1738	100%	0.0	99.58%	2759	MN544647.1
Sri Lankan cassava mosaic virus isolate SLCMA_A segment DNA-A, complete sequence	Sri Lankan cass...	1738	1738	100%	0.0	99.58%	2759	KT861468.1
Sri Lankan cassava mosaic virus isolate Chanthaburi segment A, complete sequence	Sri Lankan cass...	1738	1738	100%	0.0	99.58%	2758	MW854732.1
Sri Lankan cassava mosaic virus isolate Phetchaburi segment A, complete sequence	Sri Lankan cass...	1738	1738	100%	0.0	99.58%	2758	OM715155.1
Sri Lankan cassava mosaic virus isolate Buriram segment A, complete sequence	Sri Lankan cass...	1738	1738	100%	0.0	99.58%	2758	OM715154.1

Feedback
