# Supplementary material for: Analysis of proteomic changes in cassava cv. Kasetsart 50 caused by Sri Lankan cassava mosaic virus infection
Source: BMC Plant Biol. 2022 Dec 10;22:573. doi: 10.1186/s12870-022-03967-1 (PMC9737768; doi:10.1186/s12870-022-03967-1)
Supplement: Supplementary file 4 — Additional file 4. [file 12870_2022_3967_MOESM4_ESM.pdf]

Table S1 Primers used for RT-qPCR

No.	Primer name	Gene name	Forward primer 5'→3'	Reverse primer 5'→3'
1	A0A2C9WMD1_Catalase	Catalase	TTC GCC CCT TGC CAT GAA CA	TGA GAA CAC CAT GCT TCC AGC
2	A0A2C9V5Q3_TIR	Rpv1	CAT TTG CGA GCA TTG CCA GG	TCT CGA AAT ATA GGA TGC ACT CCA C
3	A0A2C9W2D3_ Occludin_ELL	Dentin sialophosphoprotein	GGG TCC TGG ATC TGC TCC CC	TGC GGC CAA TTC ACC TTC CT
4	A0A2C9WG55_ DCD	Uncharacterized transcript variant X5	TTT CGG CCA TTA GCG AGG GT	ACA TTG TCG TTG TTA GGC TTT GGC
5	A0A2C9UBJ8_ BEACH	Gfs12	GCC AAG ATC CTT CCA AGG TCAG	TCT TCT GTC ACC ATG CAG CAA
6	A0A2C9VQZ7_Galectin	Hydroxyproline O-galactosyltransferase GALT6	TGT GTC CTC TGA CAT TGC ATG G	AAC TGC TCC ACC CAC ATT CC
7	A0A199UBY6_ Cupin type-1	Vicilin-like seed storage protein At2g18540	CCT CCA GGC ATA GTG CAC GC	TTT GTC GCC AGC GAG AGC TT
8	A0A2C9V722_ NB-ARC	Protein RGA1	CGC TGC TAC TGT TTC TGC AGG T	TCT GAG TGT GAA CTG CGG TGA
9	A0A2C9U515_ Protein kinase	L-type lectin-domain containing receptor kinase	TTT CGG CCT AGC TCG GAT GC	CAC GAC CAC TGC GAA CCA CC
10	A0A2C9V7G1_ Receptor-like serine /threonine-protein kinase	G-type lectin S-receptor-like serine/threonine-protein kinase SD3-1	TGC TTC GGG CCA GTT ACA GC	AGG ACA GCG CTG AGG TTG GA
11	A0A2C9W892_ Serine/threonine-protein phosphatase	Serine/threonine-protein phosphatase PP2A-2 catalytic subunit	TGT GTG TGG GAA TTC GAA GAG AGT	TCG AGA TCC CTT CGA GTT TTG CT
12	A0A2C9VHG8_ LRRNT_2	Receptor-like protein 9DC3	AAG CTC CAC GGT TTG GTG AA	TCT GCA GGC AAA GGT CCA CT
13	A0A2C9ULD8_ HMA	Uncharacterized LOC110600459	GGG CTT GCT CTA GAG ATG GCT T	ACT TCC TGG ACA AGA GGG ACT GA
14	A0A2C9VG49_ AT-hook motif nuclear-localized protein	AT-hook motif nuclear-localized protein 19	CCT TGA GGG ATA CGG ATG GGC	CCA GAC GGT GAG ACG AAT CCC
15	A0A0M4FEU9_ NAC transcription factors 70	NAC transcription factors 70	AGG TCG TGC ACC CAA TGG AA	GCT CGA CAG ACC ACC CAT CC
16	A0A0M4FSG8_ NAC transcription factors 54	NAC transcription factors 54	GGA GGA AAT GGC ACC TGG GTT	AGC CGT GTT TAG GTC GTC TCT
17	A0A0M4G3M4_ NAC transcription factors 6	NAC transcription factors 6	AGC CTT CCA CAT CAC CAG GC	GGT GGT GTT GCT GGA TTT CGT
18	A0A0M5J8Q6_ NAC transcription factors 22	NAC transcription factors 22	ACT CGG GTG CTC AAA AGC CA	ACG GTC GTC GAT CTC TGG CA
19	A0A0M5JAB4_ NAC transcription factors 35	NAC transcription factors 35	GGT GTG CTT CGG CTG CCT C	TTG AAG CAG GCA AGG GGC AAG
20	A0A140H8T1_ WRKY transcription factor 77	WRKY transcription factor 77	GCA GCA GCT TCC AAT CAC GC	GTC CAC TGC CAT GGG TGA CG
21	A0A140H8T7_ WRKY transcription factor 83	WRKY transcription factor 83	AGG GAA AGG GCG GCA AGA GA	CAT CAG CTG AGA GCA ACC CAT CA
22	A0A199UCA0_ NAC	NAC transcription factors 76 mrna	GGG ATC TCC CAG GCA AGG CT	CCC TGC TTC AGT GGC TCG AT
23	A0A251J9E4_ Peptidylprolyl isomerase	Peptidyl-prolyl cis-trans isomerase FKBP53	ACC GCC AAT GAA CTT GGG GA	CCG ACC CAA CTG CAA GCT CC
24	A0A251JAR1_ DDT	Ddt	ACT TGC TCC AGG CTC TGG TTT	AAC TAC GGG GCT TGA ACA TCA T
25	A0A251JD02_ MBD	Methyl-cpg-binding domain-containing protein 11	CGG GGA GGA AAT CAC TGG CA	GTG TCT CAC CAG TGC CCC AA

No.	Primer name	Gene name	Forward primer 5'→3'	Reverse primer 5'→3'
26	A0A2C9U173_ SET	Esculenta histone-lysine N-methyltransferase ASHR1	CGT GCG TGT CCA TGG AGG AA	TGG TGA CGA GAC ACC GTC CT
27	A0A2C9U3R6_ NAC	NAC transcription factors 61	GGT GTG CCT CCT GGT TTC CG	TCC TGT AAC TCC CAA GGC TCC AC
28	A0A2C9U574_ Bromo	Transcription factor GTE12	TGC TCG TCA ATG TTG CCT TCC C	TGA GGC ATC AGA GCC ACA CG
29	A0A2C9U966_ Mediator of RNA polymerase II transcription	Mediator of RNA polymerase II transcription subunit 11	GTG CAT GCG AGT ACC GTC CA	GGC ATC CAA TTG CGC AAG CAC
30	A0A2C9UBF9_ GATA transcription factor	GATA transcription factor 12	AAA TTG TCG CGT CCC CAG CC	CGT CTT GTC GGT GGC GCA AT
31	A0A2C9UD08_ Methyltransferase	Probable methyltransferase PMT3	CAG CTC GGA AAC AGA CCA GG	GTT GGA GCA CTT GGA CTT TGG AA
32	A0A2C9UDI4_ 16S rRNA m5C967 methyltransferase	Probable ribosomal RNA small subunit methyltransferase B	GAG GTC CGG TTG TGG AGC AC	GCA CAA CCG AAA CGC CAA GC
33	A0A2C9UKD1_ Replication termination factor 2	Replication termination factor 2	GGC GGC TGA TAT GGC ACC AG	CCT ACC AAG TGG GAG CGA CC
34	A0A2C9UNL9_ BHLH	Transcription factor bhlh155	CAC TCA TTT TGG TGC TTC CAC G	AAG AGG AGG GAG GAG AAA GCA G
35	A0A2C9UY92_ KH	A-kinase anchor protein 7	TGT GAA GTC GAA GCC TGC CA	TCT TGT TGC CCA TGC TGC CT
36	A0A2C9V2G5_ Dof-type	Dof zinc finger protein	AGC TTT TGT GGG GCT TCC CAT	TGC CAA GCT GAA CCA CCA AGT
37	A0A2C9V4D0_ PWWP	Uncharacterized LOC110624857	TGC GTG GAA CGA GGC ATC TC	GAG CAG GCC AGC CCA AAC TC
38	A0A2C9VER9_ Myb-like	Trihelix transcription factor GT-3b	AAG AGA GAG AGT TCT CAG CTG GT	GTC GAT CAT CAC GTC CAC CCA
39	A0A2C9VJF7_ Tify	Protein TIFY 10b	CCACGCCAAGCTTCTCTCCT	TGCCGCTGAAGACTTGCCATC
40	A0A2C9VLJ8_ p-aminobenzoic acid synthase	Aminodeoxychorismate synthase, chloroplastic	AAG CAG GAC CGG TGG AGA GG	GAG TGG GGC CGA GGT CTC AA
41	A0A2C9VME6_ Myb-like	Lim	GGC AGC CGG AAA CCT TCT CA	TCT GCG GCG GCA AGT TTT GA
42	A0A2C9VTN6_ DNA replication ATP-dependent helicase/nuclease	DNA replication ATP-dependent helicase/nuclease JHS1	TGG GGG TTA CTA GTC CCT TGC	AGG GTC CAA GGG ATA TTG GGA GA
43	A0A2C9VVU5_ Histone acetyltransferase	Histone acetyltransferase GCN5	GCC TGC TTG AGC TGT GTG CT	GGC GAA CAC GTC ATC TCC GA
44	A0A2C9VWW7_ LIM zinc-binding	Protein DA1	TCA GAT CGC CAT TAC CAC GGA	AGG GAC AAT GCA ATA GCA CGA
45	A0A2C9W5N6_ Methyltranfer_dom	Protein RRNAD1	TGT TGG TTC TGG CCA GGG TT	TTA TCC GCT CAG CGC GTG TC
46	A0A2C9WBZ1_ BHLH	Transcription factor BEE 1	CCA CCA CCA ACA ACC ACC ACC	TCG AGG TGG AAG TGA CAG CG
47	A0A2C9WJL4_ HTH myb-type	Transcription factor HHO5	GCT TGA AGG CGG AGG AGG TC	TCA AAG GTG GAA CTC TGC ATG TTT
48	O49169_ Elongation factor 1-alpha	Elongation factor 1-alpha	TGC GCC AGA CTG TTG CTG TT	CGA ACC CTG CGT TCA CTT TCC T
49	A0SVL8_ 1-amino-cyclopropane-1-carboxylic acid oxidase	1-amino-cyclopropane-1-carboxylic acid oxidase mrna	ATG GGT CCA GGG GTC CAA CC	AGC GTC TGT GTG GGC TCT GA
50	O49893_ Alpha-hydroxynitrile lyase	Alpha-hydroxynitrile lyase	GCG GCA TTG ACC CAA GGC A	CCC TGC ACA GCT CTC ACC AA
51	A0A2C9UX68_ Annexin	Annexin-like protein RJ4	GAA CCC TCG TTG CCC CTG. C	AGC CTT CTC ATT GGT CCC CC

No.	Primer name	Gene name	Forward primer 5'→3'	Reverse primer 5'→3'
52	A0A2C9WQ86_ BAG	BAG family molecular chaperone regulator 6	CAC GAG GGT GGG CAA TCG AC	TCC GTT GGC TGC TGT TCT GC
53	A0A2C9WD07_ Catalase	Catalase isozyme 1	AGG CCA ACC ATG TGA TGA GCA	TGC CAC AAA ACT GCA ATG TCC T
54	A0A2C9UUL9_ CCHC-type	Uncharacterized LOC110607833	CGG TCG TTT GCA CAT TCG GG	TTC CGT CAA AAC ACG CCC CA
55	A0A2C9VMF9_ Clp R	Protein SMAX1-LIKE 8	TTG TTG CCG GCG ATG CCT AC	CGC GCT ACA CTC ACC GCT TC
56	A0A2C9U930_Formate dehydrogenase, mitochondrial (FDH)	Formate dehydrogenase, mitochondrial	TCA CTG TAG CTC TCG ACT CTC CA	AGA TGA GGG GAA AGC GCG AA
57	A0A2C9UKE3_GUB_WAK_bind	Uncharacterized LOC110600351	GCC TGG CGG ATG GTC TTA CG	GTA CCA TAC CCG CAG GTG CC
58	A0A2C9WN68_HSF_DOMAIN	Heat stress transcription factor B-4d	AGC CAG TTC CTG CAC CTT TCT	CGG AGG CCT ACG CAC AAC AA
59	A0A2C9WIP6_LEA_2	NDR1/HIN1-like protein 6	TGG TGC CTC CTG GTT CAG CA	CCG TGG ATT GCT GCC GGA AA
60	A0A2C9V3F4_Peptidase_M28	Reticulum metallopeptidase 1	GCC AAA CTG GTG TAG TCG CT	TGA CTG GTC GAC GGT TTC GAT
61	A0A2C9UEX5_Peroxidase	Peroxidase 15	TGC AAG CAG GGC TCA AGC TA	CCA AAC GTA TGA GCG CCG GA
62	A0A2C9VL88_PMR5N	Protein trichome birefringence-like 33	TGT TGT GGA ATT GGG AAG GAG GA	TGC TGT GAA TTT CCA TTG CAA CA
63	A0A251KQD0_Rab-GAP TBC	BC1 domain family member 2B	TCA GCT CCC CGC AAT TCA CT	CCT CGG CTC AAT ACT CCT GCT
64	A0A2C9VPT1_Reticulon-like protein	Reticulon-like protein B14	CTC ATC ATG CCG AGG TGG GG	AGA GAT CTC CGG TGG CCG AA
65	A0A2C9UNE1_S-(hydroxymethyl) glutathione dehydrogenase	Alcohol dehydrogenase class-3	TGC GTG CTT AGC GTC CAT GT	TCC ATA AAA CAC GAT CAA GGC CC
66	A0A2C9WR35_S-acyltransferase	Protein S-acyltransferase 18	TCC TCG GGC TCT TCC TTG GG	GGA TCG ATG GCA GTG CAC CTA
67	A0A2C9V533_SHSP	18.1 kda class I heat shock protein	TCC CCT CAA CAG AGG ACC CC	TCA CGT GCG ATG CTG GTG TT
68	A0A2C9U854_SPRY	Heterogeneous nuclear ribonucleoprotein U-like protein 1	ACG ACG CTC CGT CCA TTG AT	CGA ACT CGC GTT TCG AGG AC
69	A0A2C9WKA1_START	Uncharacterized LOC110617309	TCT CCA AAT CGA CTT GCA TTT TGT T	TGG CTG CCA TCA TTG AAC GTA A
70	A0A2C9WFP1_Thioredoxin	TPR repeat-containing thioredoxin TTL1	TGG CCT AGC TGG CAC CTC TC	GCT GCT TGC TGA GCT GCT GAT
71	A0A2C9URT2	C2H2-type domain-containing protein	ATG CCT TGG CGG AGG AGA GT	GAG CCA CTG GTG GGG CTA CT
72	A0A2C9W1N8	Protein FAR1-RELATED SEQUENCE	AGC CAG CGA TTC CTT GGT CG	GGG GGC TTC TGC CAT CTG TC
73	A0A251KDl2	Peptidyl-prolyl cis-trans isomerase	ACC CGA AAC CTC GGT CAT CA	GAG GTA GCA GCT GGA GAC GC
